# Quality Control and Performance of HIV Rapid Tests in a Microbicide Clinical Trial in Rural KwaZulu-Natal

**DOI:** 10.1371/journal.pone.0030728

**Published:** 2012-01-30

**Authors:** Nina von Knorring, Mitzy Gafos, Motsei Ramokonupi, Ute Jentsch, the MDP Team

**Affiliations:** 1 Africa Centre for Health and Population Studies, University of KwaZulu-Natal, Mtubatuba, South Africa; 2 Wits Reproductive Health and HIV Institute, Johannesburg, South Africa; 3 Contract Laboratory Services, University of the Witwatersrand Health Consortium, Johannesburg, South Africa; Blood Systems Research Institute, United States of America

## Abstract

**Background:**

Quality control (QC) and evaluation of HIV rapid test procedures are an important aspect of HIV prevention trials. We describe QC and performance of two rapid tests, *Determine™* and *Uni-Gold™* used in a microbicide clinical trial in rural KwaZulu-Natal, South Africa.

**Methods/Results:**

Internal QC of both HIV rapid tests was conducted at the trial site using a Uni-Gold control kit (*Uni-Gold™Recombigen® HIV*). Both assays produced the expected results for a total of 4637 QC tests. Study participants were tested for HIV at screening and, if enrolled, at regular time points throughout the study. Positive or discordant results were confirmed by a double HIV immunoassay testing strategy at a local laboratory. Overall, 15292 HIV rapid test were performed. Sensitivity and specificity of Determine was 98.95% (95% CI: 97.72–99.61) and 99.83% (95% CI: 99.70–99.91) respectively [positive predictive value (PPV) 97.91% (95% CI: 96.38–98.92)], for Uni-Gold it was 99.30% (95% CI: 98.21–99.81) and 99.96% (95% CI: 99.88–99.99) respectively [PPV 99.47% (95% CI: 98.46–99.89)].

**Conclusions:**

The results suggest that a Uni-Gold control kit can be used for internal QC of both Uni-Gold and the HIV-1 component of the Determine rapid tests. Both rapid tests performed proficiently in the trial population.

## Introduction

The Africa Centre for Health and Population Studies was one of six research centres participating in the Microbicides Development Programme (MDP) 301 phase III clinical trial [1]. The trial evaluated the effectiveness and safety of PRO2000/5 microbicide gel in the prevention of vaginally acquired HIV infection. The trial results and details of HIV seroconversion have been presented elsewhere [2], [3]. The Africa Centre is situated in the predominantly rural Umkhanyakude district of northern KwaZulu-Natal where HIV prevalence is 27% in female residents aged 15 to 49, peaking at 51% in 25 to 29 year old women [4], [5]. HIV incidence among women aged 15 to 49 remains high (4.4 per 100 person-years) [6]. Trial eligibility criteria required women to be HIV negative at screening. Two rapid tests were used to determine HIV status: *Determine™HIV-1/2* (Abbott Laboratories®, Tokyo, Japan, now marketed by Inverness Medical as *Alere Determine™*) and *Uni-Gold™HIV* (Trinity Biotech, Ireland). We evaluated a Uni-Gold control kit (*Uni-Gold™Recombigen® HIV*) comprised of HIV-1 positive and negative serum on Determine rapid tests. The kit is available for internal quality control (QC) in conjunction with the Uni-Gold rapid test. There is no dedicated control kit for the Determine test and no evidence in the literature of a previous evaluation of the Uni-Gold control kit for use with other HIV rapid tests. We also assessed the performance of both HIV tests in our trial population as some rapid tests show geographical variation of performance or vary when used in the field by non-laboratory staff [7], [8]. The data provided by the test manufacturers shows a sensitivity and specificity on serum and plasma specimens of 99.91% (95% CI: 99.51–100) and 98.16% (95% CI: 97.49–98.69) respectively for the Determine rapid tests and a sensitivity and specificity of 100% (95% CI: 95.5–100 and 97.9–100) for Uni-Gold on whole blood. The Determine rapid test has previously been evaluated in primary health and community clinics in South Africa [7], [9] and various settings in other countries on the continent showing sensitivities ranging from 60.5% to 100% and specificities between 98.9% and 100% [10]–[12]. In a further study conducted in South Africa, sensitivity and specificity of the Determine assay were 100%, for the Uni-Gold assay they were 97% and 100% respectively when performed by trained technologists in a laboratory setting [13].

### Ethics statement

Approval was obtained from the Biomedical Research Ethics Committee, University of KwaZulu-Natal (approval number T111/05). Written informed consent was obtained from all participants involved in the study.

## Methods

Both HIV rapid tests have a procedural control, or control line, included in the test. In addition, internal QC of both rapid tests was performed daily by nurses or HIV counsellors in compliance with Good Clinical Laboratory Practice guidelines using the Uni-Gold HIV-1 negative and positive control serum kit. This was done to ensure that the assays were correctly identifying HIV-1 antibody negative and positive samples. The procedures were recorded on QC worksheets and data from all worksheets completed at three MDP trial clinics were analysed to verify assay accuracy (March 2006 to August 2009). Study volunteers were tested on whole blood (finger prick) at screening and, if enrolled, at 12, 24, 40 and 52 weeks after enrolment using the two rapid tests in parallel. The tests were conducted at the clinics by trained counsellors according to manufacturer's instructions. Positive or discordant results on either test were confirmed by a double HIV immunoassay (IA) parallel testing strategy at the local virology laboratory [first line: *Vironostika® HIV-1 Microelisa System* (Biomérieux, Durham, North Carolina, USA), used from March 2006 to December 2007 when discontinued by manufacturer, then from January 2008 to August 2009: *SD BIOLINE HIV 1/2 3.0* (Standard Diagnostics, Inc. Kyonggi-do, Korea); second line: *Genetic Systems™ rLAV EIA* (Bio-Rad Laboratories, Redmond, Washington, USA)]. Venous blood was collected into SST and EDTA tubes at the study clinics and processed within 24 hours of drawing. Serum obtained from the SST was centrifuged within 6 hours of collection and stored at minus 80 degrees Celsius. EDTA tubes were centrifuged and plasma and buffy coat were obtained for storage at minus 80 degrees Celsius after a plasma aliquot was allocated for IA testing. In addition, 5% of all negative samples were randomly re-tested for the duration of the study by the local laboratory. This was defined as the external QC procedure. Sensitivity and specificity of the HIV rapid tests and the 95% confidence interval around the estimate were calculated in STATA by producing summary statistics for diagnostic tests compared to true disease status [14]. The calculations were based on the total number of true positives (HIV positive rapid confirmed positive on IA), false positives (HIV positive rapid found negative on IA), false negatives (HIV negative rapid found positive during random 5% QC) and true negatives (dual negative HIV rapids). These results were then compared to the data provided by the test manufacturers.

## Results

The Africa Centre MDP site screened 1775 women [mean age 33.4 (SD 11.1), median age 32 (IQR 23–43), ranging from 17 to 75 years] of which 1177 were enrolled. In total 4637 internal QC tests were conducted at the clinics on both HIV rapid tests using the Uni-Gold QC kit (n = 2306 with positive serum, n = 2331 with negative serum). Both assays produced the expected results for all QC tests. Overall, 15292 HIV rapid tests were performed on 1775 trial volunteers. Dual IA confirmation of positive and discordant rapid tests is shown in Table 1. Sensitivity and specificity of Determine was 98.95% (95% CI: 97.72–99.61) and 99.83% (95% CI: 99.70–99.91) respectively [positive predictive value (PPV) 97.91% (95% CI: 96.38–98.92)], for Uni-Gold it was 99.30% (95% CI: 98.21–99.81) and 99.96% (95% CI: 99.88–99.99) respectively [PPV 99.47% (95% CI: 98.46–99.89)]. It is important to note however, that double negative rapid results were accepted as HIV negative and not confirmed by IA (unless randomly selected for external QC), we have therefore not presented the negative predictive value and this might have led to an over-estimation of test sensitivity. The negative samples selected for external QC were all confirmed negative at the local laboratory.

**Table 1:** IA† results of positive and discordant HIV rapid tests.

Rapid test	IA pos	IA neg
Determine		
Negative (n = 7077)	6	7065*
Positive (n = 569)	563	12
Uni-Gold		
Negative (n = 7077)	4	7074*
Positive (n = 569)	565	3

† Concordant results on two IAs.

*Only discordant negative rapid tests retested by IA.

## Discussion

These results suggest that the *Uni-Gold™Recombigen® HIV* QC kit is useful as an internal quality control tool in a clinical setting not only for the Uni-Gold assay for which it is certified, but also for the Determine (HIV-1) rapid test. Further evaluation would be necessary to assess whether it is adequate for quality control of the HIV-2 component of the Determine rapid test. The Determine assay demonstrated a slightly lower sensitivity and a statistically significant higher specificity compared to the data provided by the manufacturer. Sensitivity and specificity of the Uni-Gold test were marginally lower. Overall, both Determine and Uni-Gold performed proficiently under field conditions in the trial area [15].

The authors would like to acknowledge the contribution of Johannes Viljoen, head of Africa Centre Virology Laboratory, and all trial participants and MDP staff, especially Hlengiwe Ndlovu, Portia Mutevedzi and Yael Hoogland. We also thank the trial sponsors and MDP team at the Clinical Trials Unit of the UK Medical Research Council.
